# Effects of Social Distancing During the COVID-19 Pandemic on Anxiety and Eating Behavior—A Longitudinal Study

**DOI:** 10.3389/fpsyg.2021.645754

**Published:** 2021-06-01

**Authors:** Fernanda da Fonseca Freitas, Anna Cecília Queiroz de Medeiros, Fívia de Araújo Lopes

**Affiliations:** ^1^Post-Graduate Program in Psychobiology, Department of Physiology and Behavior, Federal University of Rio Grande do Norte, Natal, Brazil; ^2^Health Sciences College of Trairi, Federal University of Rio Grande do Norte, Santa Cruz, Brazil; ^3^Department of Physiology and Behavior, Federal University of Rio Grande do Norte, Natal, Brazil

**Keywords:** COVID-19, social isolation, confinement, dietary behavior, mental health, human evolution

## Abstract

As social animals, humans need to live in groups. This contact with conspecifics is essential for their evolution and survival. Among the recommendations to reduce transmission of the new coronavirus (SARS-CoV-2) responsible for COVID-19 are social distancing and home confinement. These measures may negatively affect the social life and, consequently, the emotional state and eating behavior of individuals. We assessed the impact of the COVID-19 pandemic on the anxiety, premenstrual symptoms, and eating behavior of young women. Data collection was conducted in person (prepandemic—from March to December 2019) and online (during the pandemic—August 2020). A total of 71 participants, average age of 21.26 years (SD = 0.41), took part in the study. Trait anxiety during the pandemic was significantly lower than in the prepandemic period. Investigation of the “anxiety/stress” symptom of the Premenstrual Symptoms Screening Tool (PSST) revealed that this symptom was more severe before the pandemic. There was a decline in the desire for sweet and fatty foods during the pandemic. However, craving for traditional foods rose significantly in the same period. Uncontrolled and emotional eating were significantly lower during the pandemic. The results suggest that the pandemic may have had a positive impact on anxiety and eating behavior of the participants, which may be due to differences between urban and rural populations and the latter living with their families. These findings are important for raising a discussion regarding the effects of the current environment on the regulation of cognitive and dietary adaptations.

## Introduction

As social animals, human beings benefit from contact with co-specifics, since living in a group is an important strategy used by our ancestors to overcome daily challenges, which resulted in cognitive adaptations that support sociability (Dunbar, 2008, 2020; Lopes et al., 2018). Once basic needs are met, a more significant factor for the subjective and emotional well-being of most people seems to be good relationships with friends and family. In addition, the size and quality of a person’s social network also have a positive influence on mental health (Dunbar, 2017; Kenrick and Krems, 2018).

Similar to how our species evolved to live in groups, another clearly important psychological mechanism is activated in this context. The risk of dying from a contagious disease was a real and significant threat to our ancestors. However, a behavioral immune system, that is, a set of specific behaviors, may help us avoid diseases (Schaller and Park, 2011; Griskevicius and Kenrick, 2013; Ackerman et al., 2020). SARS-CoV-2 is a new coronavirus responsible for coronavirus disease 2019 (COVID-19) (Parlapani et al., 2020). In order to reduce virus transmission, social distancing and home confinement were recommended by health authorities and governments (Lakhan et al., 2020; Parlapani et al., 2020; Tang et al., 2020). However, distancing measures, even when added to activation of this behavioral immune system, may have a negative effect on the social life and emotional state of individuals (Ackerman et al., 2020; Akdeniz et al., 2020; Cornine, 2020; Di Renzo et al., 2020; Ingram et al., 2020; Shigemura et al., 2020; Volino-Souza et al., 2020; Wang et al., 2020; Zhou et al., 2020).

Thus, in addition to the protective recommendations against COVID-19, the fear of contracting the virus, and subsequently dying from it, uncertainty about disease control and vaccine availability, daily routine interruptions, economic loss, and constant exposure to negative news are factors considered highly responsible for the surge in mental health problems, such as anxiety (Akdeniz et al., 2020; Cornine, 2020; Di Renzo et al., 2020; Ingram et al., 2020; Kang et al., 2020; Ozamiz-Etxebarria et al., 2020; Shigemura et al., 2020; Volino-Souza et al., 2020; Wang et al., 2020; Zhou et al., 2020). In university students, anxiety symptoms have also been caused by concern about delays in academic activities and future job prospects (Akdeniz et al., 2020; Cornine, 2020; Zhou et al., 2020).

In principle, anxiety is a response that arises naturally as individuals are exposed to some everyday situations, serving as preparation for future events (Nesse, 2019a). However, when the response to these situations is no longer adequate (adaptive), anxiety can be pathological and present itself as a mental disorder, known as anxiety disorder. What differentiates adaptive anxiety from anxiety disorders are fear, emotional reaction, and excessive behavioral disturbances, which exist without adequate or proportional external stimulation to explain them and generally persist beyond the periods considered appropriate (Braga et al., 2010; American Psychiatric Association, 2014).

From an evolutionary point of view, it is necessary to consider that the demands of the current environment differ enormously from those in which the set of human development adaptations was selected. Thus, it is possible that an incompatibility between the evolved adaptation and the current environment favors the occurrence of these mental disorders, that is, the current environment does not drive the evolved mechanism in the way predicted (Kennair, 2003).

With respect to anxiety in women, in addition to the possible changes caused by the pandemic, the menstrual cycle also influences neurological and psychological functions. The hormonal variations that occur during the menstrual cycle, more specifically, the decline in estrogen and increase in progesterone that take place in the luteal phase or premenstrual period, may affect brain function, cognitive processes, emotional state, and appetite, among others. The symptoms associated with this phase can be variable or inconstant and generally include the following emotional signs: anger, irritability, anxiety, lack of concentration, and changes in mood and eating behavior (Johnson et al., 1995; Nissar et al., 2008).

Concerning the last point, stress and anxiety caused by social distancing, home confinement, the routine acquired during the pandemic, and partial or total loss of income (De Backer et al., 2021) may also influence eating behavior, having a negative effect on the amount and quality of the food consumed and access to it (Ammar et al., 2020; Cherikh et al., 2020; Górnicka et al., 2020; Naja and Hamadeh, 2020; Cummings et al., 2021). However, the psychological suffering caused by COVID-19 can also be related to positive eating behavior, as preparing food potentially works as an activity to relieve stress (Mosko and Delach, 2020; De Backer et al., 2021).

Many characteristics acquired by human beings through natural selection have reflected on dietary quality. Selective pressure has resulted in the foods obtained being composed of essential nutrients able to rapidly meet their nutritional needs. However, despite the maintenance of adaptive predispositions, over time, the reason for food intake was no longer exclusively to meet nutritional needs because current life conditions (such as the development of a series of diseases) have emerged (Zucoloto, 2011; Lopes et al., 2018; Nettersheim et al., 2018; Rantala et al., 2019).

Current life conditions that have a negative effect on mental health, causing stress and anxiety, can also be responsible for dietary changes (Ammar et al., 2020). In order to regulate and reduce negative emotions, these situations are generally associated with unhealthy foods, since due to the interaction between some foods and the central reward pathways, there is a propensity to desiring and consuming “tasty” foods with high levels of sugar, fat, and calories (Wardle et al., 2000; Sominsky and Spencer, 2014; Ingram et al., 2020). In line with this information, some research has observed an increase in total caloric intake (Ismail et al., 2020) and in candy intake (Deschasaux-Tanguy et al., 2020; Górnicka et al., 2020; Ismail et al., 2020; Cummings et al., 2021) during home confinement caused by COVID-19. However, Rodríguez-Pérez et al. (2020) described that home confinement promoted a low intake of sweets by the participants in their study.

In studies on eating behavior, the term food craving has been used to describe an “intense desire to eat a certain type of food” (Rogers and Smit, 2000) and is more associated with eating foods with high sugar content. The foundation for the strong preferences that many animals have for sweet foods results from their need to identify sources of metabolic fuel, especially glucose. In this respect, energy that is more readily available in food containing glucose would allow the brain to react to signals and symptoms caused by anxiety. However, the degree of sweetness does not provide quantitative information on glucose content, that is, energy (Beauchamp, 2016). Thus, the primary function of preference for sweets may lie in the flavor of these foods, since the sensation of sweetness is generally associated with a pleasant experience or reward, not necessarily proportional to the glucose content present in the food (Beulens et al., 2004; Beauchamp, 2016).

It is noteworthy that in ancestral environments, sweet flavors were associated with fruits, yams, and honey. Thus, the preference and ingestion of foods considered sweet were not considered harmful to health. In this perspective, the sweet foods currently desired by humans can be considered evolutionarily incompatible, as they are produced with large amounts of processed sugars and lacking nutrients, and the physiological mechanisms that involve insulin and glucagon have not evolved to repeatedly metabolize abnormally large amounts of sugar (Li et al., 2018).

This trend to eat certain foods, excessively or not, which manifests itself as a response to some emotional states, is called emotional eating behavior or emotional eating (Ouwens et al., 2009; Serin and Koç, 2020). Considering anxiety during the premenstrual period and its relation with eating patterns, the preference/desire and increase in sweet food consumption that occur during this time (Gingnell et al., 2012) may be related to an attempt to modulate anxiety as a symptom attributed to the luteal phase of the menstrual cycle (Bernardi et al., 2005). In addition, from an evolutionary perspective, the desire and intake of caloric foods in the premenstrual period (reflecting proximal aspects, according to Tinbergen, 1963; Nesse, 2019b) may be due to the increased metabolic expense to prepare the body for reproduction (distal aspects— Tinbergen, 1963; Nesse, 2019b). The metabolic rate in the follicular phase is approximately 7% lower than in the luteal phase of the menstrual cycle (Strassmann, 1999).

In light of the above, the aim of the present study was to assess the impact of the pandemic caused by COVID-19 on anxiety, premenstrual symptoms, and eating behavior in young women. Our expectation was that the pandemic was related to an increase in the levels of anxiety and premenstrual symptoms and that it had an impact on eating behavior, intensifying emotional eating and the craving for certain foods, especially sweets.

## Materials and Methods

### Participants and Procedures

The sample was composed of university students from rural Rio Grande do Norte state (RN), northeastern Brazil. The study occurred in two stages, one in person and one online. During the first stage, volunteers were recruited through personal contact and advertising the study on posters, in classrooms, and other common areas of the university. The second stage was conducted on the internet using the Google Forms platform and the link was shared through WhatsApp and by email (according to information reported by the participants in the first stage). The study is in accordance with Brazilian National Health Council and the Declaration of Helsinki and was approved by the Research Ethics Committee for the Faculty of Health Sciences of Trairi of the Federal University of Rio Grande do Norte (Protocol number CASE: 91161718.0.0000.5568; Research authorization: 2.830.540).

**FIGURE 1:**
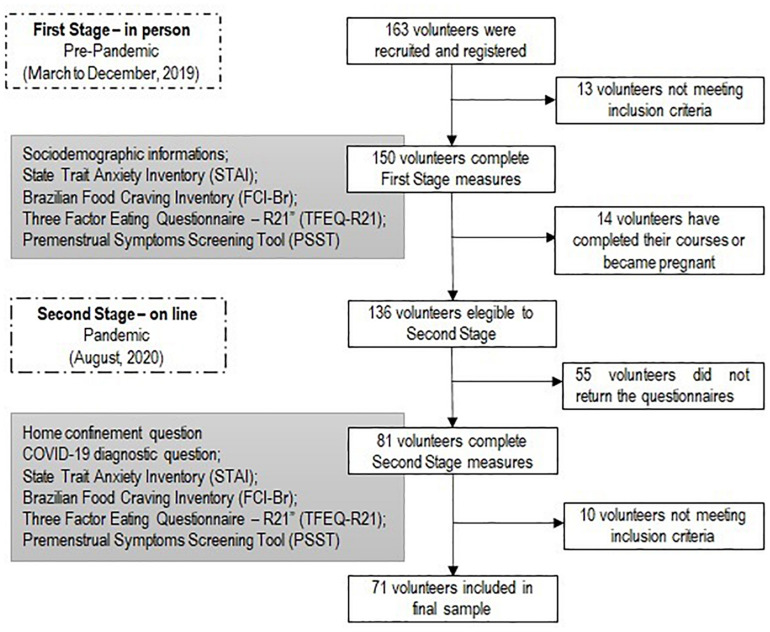
Diagram for the participants’ recruitment and inclusion in the study.

Included were volunteers aged 18 years and older who gave written informed consent to participate. Students using anxiolytics or antidepressants (including natural) were excluded from the study. The in-person stage, which occurred from March to December 2019 (prepandemic period), included 136 participants, who responded to five questionnaires: sociodemographic, Brazilian Food Craving Inventory (FCI-Br), the Brazilian version of the Three Factor Eating Questionnaire—R21 (TFEQ-R21), State Trait Anxiety Inventory (STAI), and the Brazilian version of the Premenstrual Symptoms Screening Tool (PSST). In the online stage, conducted in August 2020 (during the pandemic), 5 months after the implementation of confinement measures in Rio Grande do Norte state (RN), 71 students responded online to the same questionnaires applied in the first stage, adding whether they had been diagnosed with COVID-19 and answering a question related to home confinement (Figure 1).

### Measures and Instruments

#### Nutritional Status Assessment

Prepandemic nutritional status was assessed using body mass index (BMI), which was calculated from the weight and height self-reported by the participants in the sociodemographic questionnaire [validation of BMI from self-reported measures, in this population, was performed in the study of Lima et al. (2018)].

#### Assessment of Eating Behavior

The FCI-Br and the Brazilian version of the TFEQ-R21 were used to assess eating behavior. The FCI-Br was validated by Medeiros et al. (2017) and was created based on the Food Craving Inventory (White et al., 2002), which categorizes and identifies foods that are most related to craving, investigating the frequency of these episodes in the previous month. The FCI-Br consists of 23 foods commonly related to craving, divided into three categories: high fat content (pizza, fried pastry, bacon, salty packaged snacks, lasagna, sandwich/hamburger, *coxinha*, and French fries), sweets (cookies; dulce de leche; chocolate; condensed milk pudding; candy such as hard candy, lollipop, and jelly beans; ice cream; *brigadeiro*; sweet pie; and cake), and traditional foods (bread, barbecue/grilled meat, *farofa*, cheese, beans/*feijoada*, and steak) (Medeiros et al., 2016a; Meule, 2020).

The TFEQ was developed by Stunkard and Messick (1985) to determine cognitive restraint (CR), disinhibition, and susceptibility to hunger in adults. The first version of the TFEQ consisted of 51 items, but later studies developed shorter and psychometrically improved versions of this questionnaire, such as the TFEQ-R21. The shorter TFEQ-R21 version, adapted and translated to Brazilian Portuguese by Medeiros et al. (2016b), was created based on the TFEQ-51, containing 21 items and assessing three eating behavior dimensions: emotional eating (EA), uncontrolled eating (UE), and CR. The EA scale measures the susceptibility of consuming foods as a response to emotional stress and negative mood states. UE behavior is the tendency to lose control overeating when feeling hungry or exposed to external stimuli (for example very tasty food), even in the absence of hunger. Finally, CR is characterized as the limitation (cognitive and self-imposed) of food ingestion in order to control body weight (Medeiros et al., 2016b, 2019).

#### Assessment of Anxiety

The State-Trait Anxiety Inventory (STAI) was used to assess anxiety. The STAI was developed by Spielberger et al. (1970) and translated and adapted for Brazil by Biaggio and Natalício (1979). The tool is used to assess relatively stable anxiety. It consists of 20 statements concerning personal feelings, where the subjects report on the frequency at which these feelings generally occur. Each statement is answered on a scale varying from 1 (almost never) to 4 points (almost always), and each volunteer obtained scores between 20 and 80 (Spielberger et al., 1970; Vigneau and Cormier, 2008; Leal et al., 2017).

#### Assessment of Premenstrual Symptoms

Premenstrual symptoms were assessed using the Brazilian version of the PSST. The PSST was developed and validated for Brazil by Câmara et al. (2016). This instrument identifies the presence of premenstrual symptoms (PMS) and premenstrual dysphoric disorder (PMDD) and is composed of 19 items subdivided into two domains. Domain I consists of 14 physical and psychological manifestations of PMDD described in the diagnostic and statistical manual of mental disorders (DSM-IV). Among the symptoms assessed in this domain is “anxiety/stress.” Domain II is composed of five items that assess the functional impact of PMS. Each item is answered according to a four-point Likert scale (0 = absent; 1 = mild; 2 = moderate; 3 = severe) (Steiner et al., 2003; Henz et al., 2018).

#### Assessment of Home Confinement

The participants answered a question about how much people were leaving home (for any purpose) during confinement, using a five-point Likert scale (0 = much less than usual; 1 = less than usual; 2 = the same, no change; 3 = more than usual; 4 = much more than usual).

### Data Analysis

Data normality and homogeneity were verified by the Kolmogorov–Smirnov and Shapiro–Wilk tests, respectively. Normally distributed variables were compared using the paired Student’s *t*-test, and for the others, the Wilcoxon signed rank test was used. The relation between variables was determined by the chi-squared test. The results were considered significant at a 95% significance level (*p* < 0.05).

## Results

A total of 71 subjects, with an average age of 21.26 years (SD = 0.41) and mean prepandemic BMI of 22.93 kg/m^2^ (SD = 0.44), indicating a predominance of eutrophic nutritional status in the evaluated group (88.7% of participants), took part in the study. Three of the students (4.3%) had been infected by COVID-19.

### Anxiety

**FIGURE 2:**
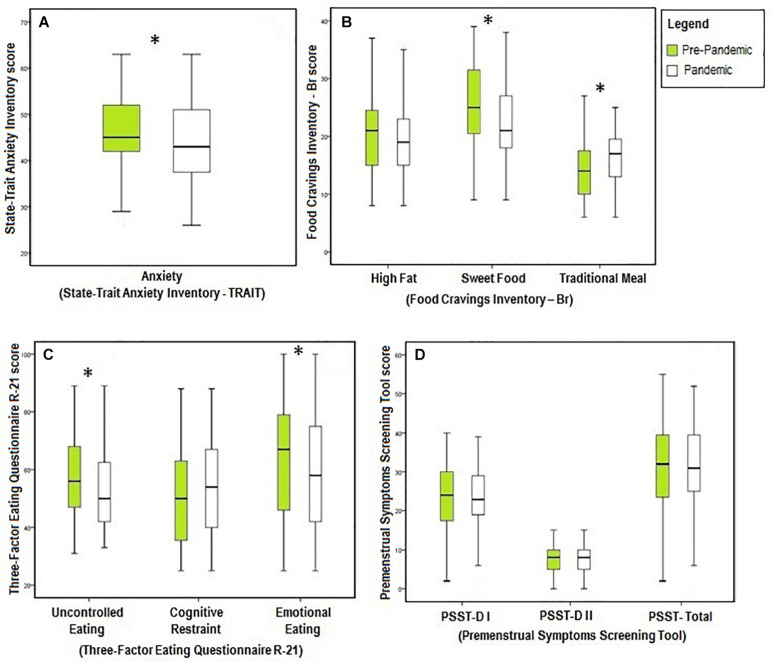
Result of the main measures observed in the study. **(A)** Trait anxiety before and during the pandemic. **(B)** Food craving before and during the pandemic. **(C)** Dimensions of eating behavior before and during the pandemic. **(D)** Premenstrual symptoms by domain and total before and during the pandemic. PSST, Premenstrual Symptoms Screening Tool; DI, domain I from PSST; DII, domain II from PSST; ^∗^*p* < 0.05.

With respect to anxiety, the STAI revealed that trait anxiety during the pandemic (44.4 ± 8.4 points) was significantly lower [*t*(70) = 2.023, *p* = 0.047, *r* = 0.23, *n* = 71] than in the prepandemic period (46.4 ± 8.7 points) (Figure 2A). It is important to underscore, reinforcing the stability of the measure, that the score remained classified as moderate anxiety despite the significant difference.

The total PSST score showed no change in the presence and severity of premenstrual symptoms between the two periods analyzed [*t*(70) = −0.013, *p* = 0.990, *n* = 71] (Figure 2D). However, the “anxiety/stress” symptoms of this tool revealed that it was more severe in the students before the pandemic [*χ*^2^(2) = 8.884, *p* = 0.012, Table 1].

**TABLE 1:** Evaluation of anxiety/stress from the Premenstrual Symptoms Screening Tool (PSST).

	Prepandemic	During the pandemic

	*n* = 71	*n* = 71
Anxiety/stress^a^*		
Absent and mild	18 (25.4%)	24 (33.8%)
Moderate	29 (40.8%)	38 (53.5%)
Severe	24 (33.8%)	9 (12.7%)

The results are expressed as *n* (%). ^*a*^Extracted from domain I from the Premenstrual Symptoms Screening Tool. *p < 0.05 in Pearson’s chi-squared test.

### Eating Behavior

Considering food craving, there was a significant decline in the desire for sweet foods during the pandemic [25.0 (20.5–31.5) versus 21.0 (18.0–27.0), *z* = −3.776, *p* < 0.001, *r* = 0.45, *n* = 71]. The craving for traditional foods rose significantly in the same period [14.0 (10.0–17.5) versus 17.0 (13.0–19.5), *z* = −3.203, *p* = 0.001, *r* = 0.38, *n* = 71] (Figure 2B).

In relation to the three dimensions of eating behavior assessed by TFEQ-R21, uncontrolled [56.0 (47.0–68.0) versus 50.0 (42.0–62.5), *z* = −2.771, *p* = 0.006, *r* = 0.33, *n* = 71] and emotional eating [67.0 (46.0–79.0) versus 58.0 (42.0–75.0), *z* = −2.229, *p* = 0.026, *r* = 0.26, *n* = 71] were significantly lower during the pandemic. Cognitive restraint increased during this time, but the difference was not significant (Figure 2C).

### Actions During the Pandemic

In relation to “outings during social distancing,” 49 (69.0%) of the participants left their home “much less than usual,” 21 (29.6%) “less than usual,” and 1 (1.4%) “more than usual.”

**FIGURE 3:**
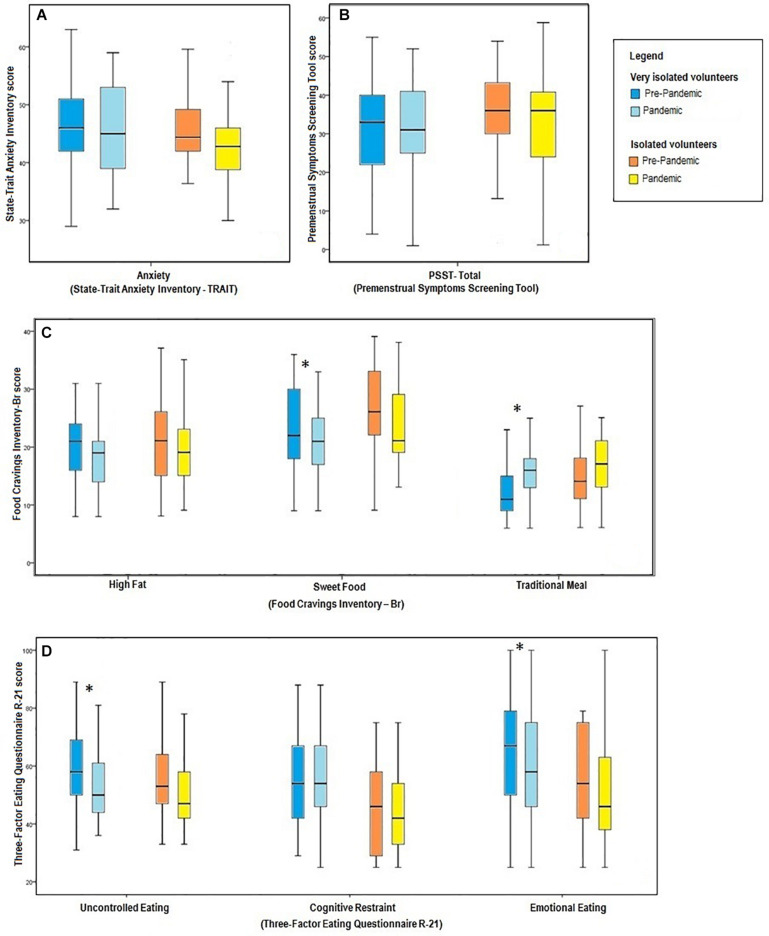
Comparison between the different measures obtained in the study, considering the confinement at home of participants during the pandemic. **(A)** Trait anxiety according to confinement at home. **(B)** Premenstrual symptoms according to confinement at home. **(C)** Food craving according to confinement at home. **(D)** Dimensions of eating behavior according to confinement at home. PSST, Premenstrual Symptoms Screening Tool; ^∗^*p* < 0.05.

As shown in Figure 3, when analyzed separately, differences were identified between the prepandemic and pandemic period only in the group that left their home “much less than usual,” which demonstrated a decline in craving for sweet foods [26.0 (22.0–33.0) versus 21.0 (19.0–29.0), *z* = −3.616, *p* < 0.001, *r* = 0.52, *n* = 49] and uncontrolled [58.0 (50.0–69.0) versus 50.0 (44.0–61.0), *z* = −2763, *p* = 0.006, *r* = 0.39, *n* = 49] and emotional eating [67.0 (50.0–79.0) versus 58.0 (46.0–75.0), *z* = −1.982, *p* = 0.047, *r* = 0.28, *n* = 49], as well as an increase in craving for traditional foods [26.0 (22.0–33.0) versus 21.0 (19.0–29.0), *t*(48) = −2.733, *p* = 0.009, *r* = 0.37, *n* = 49].

When the prepandemic and pandemic periods were compared, no significant differences were found in terms of trait anxiety (Figure 3A), presence and severity of premenstrual symptoms (Figure 3B), “anxiety/stress” symptom, craving for fatty foods, and food restraint.

## Discussion

Stressful situations observed in contemporary environment may be associated with the desire to eat foods with high sugar or fat contents, since there is an attempt to attenuate psychological stress by consuming these foods (Rantala et al., 2019). In this respect, the spread of the pandemic and the protective measures adopted, such as social isolation and distancing, would compromise mental health and lead to the intake of unhealthy foods (Wardle et al., 2000; Sominsky and Spencer, 2014; Ammar et al., 2020; Ingram et al., 2020).

Unlike the above-described expectation, the present study found that the anxiety trait remained moderate before and during the pandemic and that the “anxiety/stress” symptom was milder during the pandemic. It also revealed a decrease in craving for sweet foods and an increase for traditional foods during the same period, in addition to a decline in uncontrolled and emotional eating.

Pandemics cause concern and anxiety is a commonly observed psychological problem in these situations. Reinforcing this fact, it was reported that in China (Wang et al., 2020), Spain (Ozamiz-Etxebarria et al., 2020), Japan (Shigemura et al., 2020), Wuhan, and Scotland, COVID-19 caused mental problems in the population (Ingram et al., 2020).

However, although the fear of contracting the virus is a continuous stressor that stimulates the appetite and emotional eating mediated by glucocorticoids (Sominsky and Spencer, 2014), biological (Rantala et al., 2018), sociocultural, economic, and environmental factors may affect the mental health of individuals differently in stressful situations (Liu et al., 2020). For example, confinement can produce healthier eating habits, due to the greater time available for cooking and fewer temptations to eat harmful foods (Ingram et al., 2020; López-Bueno et al., 2020; Rodríguez-Pérez et al., 2020).

With respect to home confinement, our results corroborate these explanations in that the participants who leave home “much less than usual” during the pandemic reported a decline in craving for sweet foods, while craving for traditional foods increased, likely due to the greater exposure to this type of food.

In this respect, an electronic survey conducted in Poland (PLifeCOVID-19) revealed that 48% of those interviewed reported an increase in the consumption of home-cooked meals, attributed to the increased possibility of preparing a meal at home or more free time to take care of health during home confinement (Górnicka et al., 2020). Similarly, the COVIDiet study (Rodríguez-Pérez et al., 2020) carried out with 7,514 Spanish adults showed that during COVID-19 confinement, there was significant adherence to the Mediterranean diet (characterized by the consumption of extra virgin olive oil, whole grains, and vegetables, with low intake of sweets, red meat, and processed foods; Bach-Faig et al., 2011).

Another cross-sectional online survey carried out in 38 countries, which investigated three domains of food literacy behavior (food planning, selection, and preparation), observed that during the COVID-19 pandemic, the perception of having more time was associated with increases in planning, selecting, and preparing healthier foods for women and men and that policies for staying at home or working at home were positively related to planning and preparing healthier foods for both sexes (De Backer et al., 2021).

On the other hand, an online survey on mental health and lifestyle during home confinement (ECLB-COVID19), involving 35 research organizations from Europe, North Africa, Western Asia, and the Americas, found an unhealthy dietary pattern (type of food, uncontrolled eating, between-meal snacks, and number of main meals) during home confinement (Ammar et al., 2020). Another study with 1,012 participants from the United Arab Emirates showed a significant increase in the percentage of subjects that consumed primarily home-cooked meals, and that the foods selected were not nutritious, with the daily presence of sweets and savory snacks, which was also attributed to restricted food access (Ismail et al., 2020).

De Backer et al. (2021) also observed that staying at home was negatively associated with the selection of healthier foods for women and men. In addition, another relevant finding was a greater psychological distress observed in women during the COVID-19 pandemic and that this psychological distress was related to decreased planning, selection, and preparation of healthier foods. Among men, there was an increase in the preparation of healthier meals when psychological suffering increased. This result can be explained by the possibility that, during the pandemic, psychological suffering became a barrier to the daily cooking of women, but for men, it was an alternative to relieve stress.

It is important to underscore a significant aspect of the present study, namely that food intake was not measured, but rather eating behavior. Particularly with respect to craving, the decrease and increase in craving for sweets and traditional foods, respectively, indicate a change in the desire to eat a certain type of food, suggesting an intriguing perspective for the future eating habits of these women.

In relation to the impact of the pandemic on dietary behavior, 638 undergraduate and former undergraduate female students from King Saud University in Saudi Arabia were studied. In this survey, approximately half the women in the sample reported emotional eating (EE) during the COVID-19 pandemic. A total of 335 women (52.5%) reported low EE, while 202 (31.7%) were in the moderate and 101 (15.8%) in the high EE group. The study also demonstrated that eaters with the highest EE score had a trend to report anxiety. However, no significant association was observed between anxiety and EE. One possible explanation is that anxiety provoked by COVID-19 was an acute stressor, thereby reducing appetite and emotional eating (Al-Musharaf, 2020). It is important to emphasize that these reports were compiled over a short period of time after the onset of the pandemic.

In the United States, the study by Cummings et al. (2021) demonstrated that during the COVID-19 pandemic, American adults increased the addition of sugars to foods by 14%, but despite this increase in sugar intake, there was no increase in total food consumption in response to the stress caused by the pandemic. So, the reason for this sharp change in eating behavior may be due to the purchase of more nonperishable processed foods during this period.

Given these different results, a key factor to consider is that the participants of the present study are university students from a rural area of the state, but most come from small towns in the region and are only living in the city where the university is located for study purposes. Living outside the family home has been a factor related to poor quality of diet and emotional and anxiety disorders in undergraduate students (Papadaki et al., 2007; Zurita-Ortega et al., 2018; Cao et al., 2020). Thus, despite the protective measures and the concern about the new coronavirus, returning to their hometowns to be with their families may explain the lower anxiety and eating behaviors observed.

As humans are highly social animals, the response to the threat of infection by COVID-19 causes the desire for contact and social support, especially in relation to loved or vulnerable people, such as family members (Dezecache et al., 2020). The loss of this type of social contact can have an impact on subjective well-being (Paredes et al., 2021), with a negative response from the nervous system, since the activation of some psychological mechanisms in contexts of food sharing and other forms of cooperation so important in the evolutionary history of our species is limited (Ringer et al., 2019).

This influence seems to be supported by evidence from the Spanish study COVIDiet, which found that living at home with one’s family may be associated with a healthier diet (Rodríguez-Pérez et al., 2020). A study with 7,143 Chinese university students reported that living with families/parents during the pandemic may be a protective factor against anxiety (Cao et al., 2020). In addition, it is believed that people who frequently eat with others are happier and more satisfied with their lives, since eating socially releases endorphins, which may promote the same positive effect as that caused by physical exercise (Cohen et al., 2010; Dunbar, 2017).

One aspect that deserves attention is the opportunity we had to observe the behavior of a group outside of large urban centers. A number of factors indicate that living in small cities may also protect individuals against stress and anxiety. Most cases of COVID-19 occurred in the state capitals, resulting in greater sensitivity and vulnerability to psychosocial impacts of the pandemic on the residents of these communities. The high population densities of the capitals facilitate the transmission of the virus, producing stress due to the increased perceived risk of infection. Moreover, living in large cities may also raise the likelihood of access to communication and information. A study performed with 3,068 subjects in China, consisting of 1,928 urban dwellers and 1,140 rural residents, reported a significantly higher prevalence of mental health problems associated with the COVID-19 pandemic in urban individuals compared with those who live in rural environments (Liu et al., 2020).

In addition to the reasons explained above, the higher incidence of mental health problems in individuals living in urban environments may be due to an evolutionary incompatibility, as different from the ancestral world and small cities, urban centers are marked by a greater dispersion of families, less exposure to nature, sleep disturbances, greater sedentary lifestyle, and greater intake of processed foods (Rantala et al., 2021). As societies globalize and human-induced environmental changes increase rapidly, evolutionary mismatches are becoming increasingly prevalent (Li et al., 2018).

The protective measures implemented due to the COVID-19 pandemic may have aggravated the practice of unhealthy lifestyles, such as physical inactivity and the consumption of processed foods. In this sense, in addition to the stress and anxious symptoms caused by the incompatibility of biological and cognitive expectations between the ancestral and the contemporary environment of urban centers (Kavanagh and Kahl, 2018), the negative changes in mood can also be related to neuroinflammation caused by current and evolutionarily new lifestyles, as factors such as unhealthy eating and low or no physical activity favor the increase in the serum amount of proinflammatory cytokines (Rantala et al., 2018, 2021).

The influence of returning to their families and the fact that the study population live in small cities seem to be essential to understanding the results of the present study, since they contradict other research, which found an increase in anxiety levels among university students in several countries (Akdeniz et al., 2020; Cornine, 2020; Wang et al., 2020; Zhou et al., 2020). In our study, we recorded a reduction in anxiety trait expression. Although we are referring to a trait, and therefore, with less expectation of change, Cohen et al. (2014) argue that it is possible that there are changes in the expression of a trait, especially when we consider the nature of the situations in which the trait is expressed. Dealing with the pandemic situation has been one of the greatest experiments that humanity has been facing. We still have no way of accurately measuring the psychological effects that such a situation can have in the short, medium, and long term.

This study has several limitations due to the following factors: not investigating the economic situation and exposure to pandemic-related information of the participants; the data being self-reported, which may have introduced memory bias; and the online stage having occurred at a time when some of the social distancing measures had been relaxed (beginning of the reopening of commercial stores and nonessential services). Nevertheless, the study exhibits strong points that produced robust results. We compared data collected in the prepandemic and pandemic periods, and the sample was non-urban (less studied in other research) and composed of young women, who are more predisposed to developing mental disorders. Also, as a positive aspect, the study was carried out in a sample of the Latin American population that was little studied. Most research are conducted in North America or Western Europe (Henrich et al., 2010). Thus, in order to obtain representativeness regarding human species diversity, science needs to include more cultures (Barrett, 2020), focusing particularly on less studied populations. Still, at a time when public policies for coping with COVID-19 have been formulated and implemented quite quickly, the present study warns about the importance of studying differences between urban and rural environments. Different contexts can generate different responses to the current state of the world pandemic, reflecting the need for differentiated strategies in health care for the population.

In conclusion, our findings suggest that the pandemic may have had a positive impact on the anxiety and eating behavior of the participants, which may be due to the differences between urban and city dwellers and living with families. These findings demonstrate the importance of discussing the effects of the current environment on the regulation of cognitive and dietary adaptations, as well as emphasizing the importance of diversifying participants, since contexts in which we live are essential to understanding possible variations in the behavioral expression of our species.

## Data Availability Statement

The raw data supporting the conclusions of this article will be made available by the authors, without undue reservation.

## Ethics Statement

The studies involving human participants were reviewed and approved by Research Ethics Committee for the Faculty of Health Sciences of Trairi of the Federal University of Rio Grande do Norte under protocol number 2.830.540. The patients/participants provided their written informed consent to participate in this study.

## Author Contributions

FF, AM, and FL conceived and designed the study, conducted the statistical analysis, and wrote and revised the manuscript. FF organized the database. All the authors contributed to the article and approved the version submitted.

## Conflict of Interest

The authors declare that the research was conducted in the absence of any commercial or financial relationships that could be construed as a potential conflict of interest.
